# Supplementary material for: Effects of Group Exercise Intervention on Quality of Life and Physical Parameters in Patients with Childhood Cancer: A Systematic Review
Source: Curr Oncol. 2024 Feb 15;31(2):1035–46. doi: 10.3390/curroncol31020077 (PMC10887515; doi:10.3390/curroncol31020077)
Supplement: Supplementary file 1 [file curroncol-31-00077-s001.zip › curroncol-2843951-supplementary.pdf]

Supplementary Table S1 Terms used in the search performed on September 27, 2023

#1	"adolescent"[MeSH Terms]
#2	"child"[MeSH Terms]
#3	"schoolchild"[Title/Abstract]
#4	"kid"[Title/Abstract]
#5	"kids"[Title/Abstract]
#6	"adoles"[Title/Abstract]
#7	"teen"[Title/Abstract]
#8	"boy"[Title/Abstract]
#9	"girl"[Title/Abstract]
#10	"minor"[Title/Abstract]
#11	"youth"[Title/Abstract]
#12	"puberty"[Title/Abstract]
#13	"pubescent"[Title/Abstract]
#14	"prepuberty"[Title/Abstract]
#15	"pediatric"[Title/Abstract]
#16	"school"[Title/Abstract]
#17	"preschool"[Title/Abstract]
#18	"high school"[Title/Abstract]
#19	#1 or #2 or #3 or #4 or #5 or #6 or #7 or #8 or #9 or #10 or #11 or #12 or #13 or #14 or #15 or #16 or #17 or #18
#20	"Neoplasms"[MeSH Terms]
#21	"oncolog"[Title/Abstract]
#22	"neoplas"[Title/Abstract]
#23	"carcinom"[Title/Abstract]
#24	"tumor"[Title/Abstract]
#25	"tumour"[Title/Abstract]
#26	"cancer"[Title/Abstract]
#27	"malignan"[Title/Abstract]
#28	"hemato-oncologic"[Title/Abstract]
#29	"hematolo"[Title/Abstract]
#30	"haemato-oncologic"[Title/Abstract]
#31	"haematolo[Title/Abstract"
#32	"bone marrow transplant"[Title/Abstract]
#33	"leukemi"[Title/Abstract]
#34	"leukaemi"[Title/Abstract]
#35	"AML"[Title/Abstract]
#36	"lymphom"[Title/Abstract]
#37	"hodgkin"[Title/Abstract]
#38	"T-cell"[Title/Abstract]
#39	"B-cell"[Title/Abstract]
#40	"non-hodgkin"[Title/Abstract]
#41	"sarcom"[Title/Abstract]
#42	"Ewing"[Title/Abstract]
#43	"osteosarcom"[Title/Abstract]
#44	"orwilms"[Title/Abstract]
#45	"nephroblastom"[Title/Abstract]
#46	"neuroblastom"[Title/Abstract]
#47	"rhabdomyosarcom"[Title/Abstract]
#48	"teratom"[Title/Abstract]
#49	"hepatom"[Title/Abstract]
#50	"hepatoblastom"[Title/Abstract]
#51	"medulloblastom"[Title/Abstract]
#52	"PNET"[Title/Abstract]
#53	"retinoblastom"[Title/Abstract]
#54	"meningiom"[Title/Abstract]
#55	"gliom"[Title/Abstract]
#56	"Brain Neoplasms"[Title/Abstract]
#57	"Brain Tumor"[Title/Abstract]
#58	"Brain Cancer"[Title/Abstract]
#59	#20 or #21 or #22 or #23 or #24 or #25 or #26 or #27 or #28 or #29 or #30 or #31 or #32 or #33 or #34 or #35 or #36 or #37 or #38 or #39 or #40 or #41 or #42 or #43 or #44 or #45 or #46 or #47 or #48 or #49 or #50 or #51 or #52 or #53 or #54 or #55 or #56 or #57 or #58
#60	"Physical Therapy Modalities"[MeSH Terms]
#61	"exercise"[MeSH Terms]
#62	"exercises"[Title/Abstract]
#63	"physical activities"[Title/Abstract]
#64	"physical activity"[Title/Abstract]
#65	"physical exercise"[Title/Abstract]
#66	"physical exercises"[Title/Abstract]
#67	"exercise training"[Title/Abstract]
#68	"exercise trainings"[Title/Abstract]
#69	"exercise therapy"[Title/Abstract]
#70	"Aerobic Exercise"[Title/Abstract]
#71	"Aerobic Exercises"[Title/Abstract]
#72	"Exergaming"[Title/Abstract]

#73	"sport"[Title/Abstract]
#74	"sports"[Title/Abstract]
#75	"recreation"[MeSH Terms]
#76	"recreation therapy"[MeSH Terms]
#77	"physical fitness"[Title/Abstract]
#78	"swimming"[Title/Abstract]
#79	"fitness"[Title/Abstract]
#80	"physical education"[Title/Abstract]
#81	"physical training"[Title/Abstract]
#82	"dancing"[Title/Abstract]
#83	"pilates"[Title/Abstract]
#84	"physical intervention"[Title/Abstract]
#85	"play therapy"[Title/Abstract]
#86	"APA"[Title/Abstract]
#87	"adapted physical activities"[Title/Abstract]
#88	#60 or #61 or #62 or #63 or #64 or #65 or #66 or #67 or #68 or #69 or #70 or #71 or #72 or #73 or #74 or #75 or #76 or #77 or #78 or #79 or #80 or #81 or #82 or #83 or #84 or #85 or #86 or #87
#89	"group therapy"[MeSH Terms]
#90	"group format"[MeSH Terms]
#91	"group treatment"[MeSH Terms]
#92	"group intervention"[MeSH Terms]
#93	"pair"[Title/Abstract]
#94	"pair training"[Title/Abstract]
#95	#89 or #90 or #91 or #92 or #93 or #94
#96	"randomized controlled trial"[Publication Type]
#97	"controlled clinical trial"[Publication Type]
#98	"Randomized Controlled Trial"[MeSH Terms]
#99	"Random Allocation"[MeSH Terms]
#100	"Double-Blind Method"[MeSH Terms]
#101	"Single-Blind Method"[MeSH Terms]
#102	"clinical trial"[Publication Type]
#103	"Clin* NEAR/25 Trial*"[Title/Abstract]
#104	"Cohort Studies"[MeSH Terms]
#105	"Comparative Study"[MeSH Terms]
#106	"Cross-Sectional Studies"[MeSH Terms]
#107	"Non-Randomized Controlled Trials as Topic"[MeSH Terms]
#108	"intervention study"[Title/Abstract]
#109	"intervention studies"[Title/Abstract]
#110	"interventional study"[Title/Abstract]
#111	"interventional studies"[Title/Abstract]
#112	#96 or #97 or #98 or #99 or #100 or #101 or #102 or #103 or #104 or #105 or #106 or #107 or #108 or #109 or #110 or #111
#113	#19 and #59 and #88 and #95 and #112
